# From deposit to disposition: behavioural determinants of participation in hungary’s REpont deposit-return system

**DOI:** 10.1038/s41598-026-67353-9

**Published:** 2026-08-17

**Authors:** Sayyed Khawar Abbas

**Affiliations:** Department of Information Systems, Corvinus University of Budapest, Budapest, Hungary

**Keywords:** Deposit-return system, Consumer behaviour, PLS-SEM, Circular economy, Hungary, Institutional trust, Mixed methods, Behavioural economics, Three-component attitude model, REpont, MOHU, Value-action gap, Psychology, Psychology, Science, technology and society

## Abstract

Deposit-return systems (DRS) have acquired renewed policy urgency across the European Union following the adoption of the 2024 Packaging and Packaging Waste Regulation (PPWR, Regulation (EU) 2025/40), which obliges all member states to achieve a 90% separate collection rate for single-use beverage containers by 2029. Hungary’s REpont scheme, operational from January 2024 and administered by MOHU (MOL Hulladékgazdálkodási Zrt.) through more than 4,700 collection points nationwide, represents one of the youngest and most centralised DRS architectures in the European Union. The behavioural determinants of consumer participation in early-stage systems of this kind remain poorly understood, particularly regarding the interaction between financial incentives, multi-component psychological attitudes, institutional trust in a commercially operated concession holder, and culturally embedded recycling norms. This study employs a sequential explanatory mixed-methods design that combines a large-scale nationally post-stratification-weighted survey (*n* = 2,665) analysed via Partial Least Squares Structural Equation Modelling (PLS-SEM) with 22 semi-structured stakeholder interviews covering consumers, retail employees, and system operators. An Extended Behavioural Circular Economy (EBCE) model is proposed and empirically tested, indicating a sequential affective-to-cognitive-to-conative-to-use predictive chain (β = 0.633, 0.799, 0.268; all *p* < 0.001). Institutional trust significantly moderates the cognitive-to-use relationship (βinteraction = 0.183, *p* < 0.001), and perceived convenience exerts an independent direct effect on system use (β = 0.157, *p* < 0.001) alongside the conative-to-use path. Qualitative analysis reveals four structural participation barriers: reverse vending machine (RVM) infrastructure friction, social stigma at return points, a culturally embedded bottle-accumulation practice, and operator legitimacy concerns arising from MOHU’s petrochemical parent company. K-means cluster analysis identifies a paradoxical low-participation cohort among highly educated urban residents, consistent with the value-action gap. Post-stratification weighting via iterative proportional fitting aligns the sample with national population benchmarks on gender, age, education, and region, improving demographic alignment with Hungarian population benchmarks. The study contributes a tested five-construct behavioural model and offers evidence-informed policy recommendations for DRS design across transitioning EU economies.

## Introduction

The European Union’s Packaging and Packaging Waste Regulation (PPWR), adopted in December 2024 under Regulation (EU) 2025/40, constitutes the most decisive legislative shift in European packaging governance in over two decades. By imposing a mandatory 90% separate collection target for single-use plastic and metal beverage containers by 2029, the PPWR compels most member states to adopt deposit-return systems as their principal separate collection mechanism^1,2^ subject to derogation for states already achieving high separate-collection rates. This mandate is grounded in compelling empirical foundations: across European countries with fully operational systems, DRS collection rates consistently exceed 85%, and leading adopters, including Germany and Norway, approach or exceed 98%, compared with a global average of 81–82% for broad-scope systems^3,4^.

Deposit-return systems function through a straightforward behavioural mechanism: consumers pay a refundable deposit at the point of purchase, which is redeemed upon returning the empty container to an authorised collection point, typically through a reverse vending machine (RVM) at a retail outlet. The financial incentive is designed to overcome the transaction costs, habitual inertia, and informational barriers that suppress participation in voluntary recycling systems. Yet the empirical literature consistently demonstrates that deposit levels and infrastructure provision are necessary but insufficient conditions for sustained participation^5,6^. Psychological attitudes, social norms, institutional trust, and infrastructure convenience collectively determine whether the financial incentive translates into durable behavioural change^7,8^.

Hungary occupies a structurally distinctive position within this European landscape. Its beverage container separate collection rate stood at approximately 30–40% before the REpont launch, among the lowest in the EU and substantially below the 77% collection target required under the Single-Use Plastics Directive by 2025^9^. The REpont scheme, operational from 1 January 2024, introduced a uniform 50 HUF deposit (approximately EUR 0.13) on all eligible single-use plastic, metal, and glass beverage containers between 0.1 and 3.0 L. The system is operated by MOHU under a state-granted concession, deploying over 1,700 automated reverse vending machines (predominantly TOMRA and Envipco models) alongside more than 3,000 manual return points^10,11^. By May 2025, over 2 billion containers had been returned through the system, reflecting a rapid initial uptake^12^.

Despite this operational momentum, the behavioural architecture underlying sustained consumer participation is poorly understood. What drives some consumers to integrate REpont into weekly routines while others, including the most environmentally knowledgeable, remain infrequent users? The existing DRS behavioural literature, primarily based on mature Nordic and Western European systems, identifies attitude components, infrastructure convenience, and social norms as the primary behavioural determinants^5,13,14^. However, this literature has not examined early-stage Central European systems characterised by novel operator structures, cultural norms around bottle storage, and significant urban-rural infrastructure disparities. The role of institutional trust, in particular, has received limited formal attention in DRS research despite its theoretical centrality in environmental policy compliance literature^15,16^.

This study addresses these gaps by proposing and testing the Extended Behavioural Circular Economy (EBCE) model, a five-construct PLS-SEM framework that integrates the three-component attitude model (3CAM) with institutional trust and perceived convenience. The model is validated on a nationally post-stratification-weighted survey of *n* = 2,665 Hungarian consumers and enriched by 22 semi-structured multi-stakeholder interviews. Three research objectives are pursued: (i) to validate the EBCE model and test six directional hypotheses using PLS-SEM on weighted survey data; (ii) to identify demographic and geographic moderators of DRS participation through cluster analysis; and (iii) to illuminate qualitative mechanisms connecting attitudes to behaviour and explaining the paradoxical low-participation pattern among highly educated urban consumers.

The remainder of this paper is organised as follows. Section  2 reviews the theoretical and empirical literature. Section  3 presents the EBCE conceptual model and hypotheses. Section  4 describes the mixed-methods research design, data collection procedures, and analytical strategy. Section  5 reports quantitative and qualitative results. Section  6 discusses the integrated findings and their theoretical contributions. Section  7 presents policy implications. Section  8 acknowledges limitations and directions for future research. Section  9 concludes.

## Literature review

### Deposit-return systems: technical and environmental evidence

The empirical evidence on DRS effectiveness is extensive and convergent. European countries with mature systems achieve collection rates for covered packaging that consistently exceed 85%, outperforming municipal kerbside collection by 20–30% points1,3. The quality advantage is equally substantial: recycled PET collected through DRS contains substantially fewer contaminating particles than material from kerbside streams (by a factor of approximately nine, according to van Velzen et al.)^17^. Beyond collection efficiency, DRS implementation has been associated with litter reductions of approximately 40–60% in coastal and urban environments (Brizga et al.)^18^, a figure that should be contextualised relative to specific national rollout conditions18. Germany’s system currently achieves a return rate exceeding 98%; Norway reaches 92%; Ireland, which launched its DRS in February 2024, attained a 73% return rate within seven months, already approaching the EU’s 77% intermediate target3.

Economic assessments have historically framed DRS costs as higher than municipal EPR alternatives, but recent comprehensive analyses challenge this view. Cost-benefit analyses from Australia, New Zealand, and Sweden demonstrate net positive social welfare when litter reduction externalities, avoided municipal processing costs, and high-quality recyclate revenues are monetised^19^. Sidorczuk-Pietraszko et al.1 provide the most comprehensive evidence review to date, concluding that both scientific and empirical evidence support the EU’s policy decision: collection rates in mature DRS systems universally exceed 85%, and the incremental investment in infrastructure is recovered within 3–6 years in most EU contexts. Critically, their review identifies deposit level and infrastructure density as the primary performance-limiting variables in early-stage systems. This is not in conflict with the present study’s focus on psychological attitudes; rather, it motivates the question of how attitude-behaviour relationships are shaped by perceived system quality — which is itself a function of infrastructure provision.

### Psychological frameworks for DRS participation

The psychology of DRS participation draws on multiple theoretical traditions. Ajzen’s^20^ Theory of Planned Behaviour (TPB) identifies behavioural intention as the proximate cause of action, mediated by attitudes, subjective norms, and perceived behavioural control. Extensive meta-analyses confirm TPB’s predictive validity for recycling behaviour^21^, but critics argue it underweights affective, habitual, and moral dimensions of environmental behaviour^22^. The Value-Belief-Norm framework of Stern et al.^23^ situates pro-environmental behaviour within a value-to-action chain, emphasising moral norm activation as the proximate driver, an approach that captures the ethical motivational structure of DRS participation.

The three-component attitude model (3CAM) of Breckler^24^ offers a more differentiated theoretical lens by decomposing attitudes into cognitive (knowledge and evaluative beliefs), affective (emotional appraisal), and conative (intention and habitual readiness) dimensions. Applications of 3CAM in recycling and packaging contexts consistently show that affective and conative components are stronger predictors of habitual behaviour than cognitive evaluations^25,26^. Ráti and Maró^13^ confirmed this pattern in the Hungarian context, establishing the affective-cognitive-conative sequential mediation chain in an early analysis of REpont participation. Critically, their results suggested that cognitive knowledge about the system can exert a counterintuitive negative effect on use frequency when infrastructure shortcomings are salient, a finding that motivates the present study’s integration of institutional trust as a moderating construct.

### Institutional trust in environmental systems

Institutional trust, defined as the degree to which individuals believe an authority or organisation acts competently, fairly, and in alignment with public values^27^, has been extensively studied in risk regulation and environmental policy compliance contexts. Poortinga and Pidgeon^15^ demonstrated that trust mediates the relationship between policy knowledge and behavioural adoption, with low-trust individuals showing reduced compliance even when cognitive awareness is high. Earle and Cvetkovich’s^27^ salient value similarity model argues that trust is grounded in perceived alignment between an institution’s actions and citizens’ core values, a dimension particularly salient in the Hungarian context where MOHU’s parent company, MOL Group, is a major oil and gas producer.

In DRS contexts specifically, institutional trust encompasses at least four distinct sub-dimensions: trust in correct deposit refund processing, confidence that returned materials genuinely reach recycling facilities, belief that the operator’s mission is aligned with public environmental goals, and perceived fairness of the concession arrangement. None of these dimensions has been formally operationalised or tested in published DRS behavioural research, representing a gap in the published literature that the EBCE model addresses directly.

### Perceived convenience and infrastructure access

Perceived convenience, broadly defined as the degree to which a consumer perceives the physical and temporal costs of system use as acceptable, is consistently identified as one of the strongest practical predictors of DRS participation rates^3,5^. Reloop’s^3^ international performance analysis confirms that systems with return-to-retail models, where consumers can return containers at supermarkets they visit during regular shopping trips, achieve substantially higher participation rates than systems relying on dedicated redemption centres. In the Hungarian context, infrastructure density is a critical variable: the REpont network, while growing, had concentrated RVMs in large supermarkets at launch, leaving inner-city Budapest with insufficient coverage for its population density. Convenience incorporates not only proximity but also queue time, machine functionality, and the ease of the return process, dimensions captured by the Perceived Convenience construct in the EBCE model.

### Research gaps and contributions

Three interconnected gaps motivate this study. First, while Ráti and Maró’s^13^ quantitative study provides the most rigorous prior analysis of REpont participation, its exclusively survey-based design cannot illuminate the qualitative mechanisms, social dynamics, and operator-level constraints that jointly shape behavioural outcomes. Second, no published DRS behavioural model formally incorporates institutional trust as a moderating construct, despite strong theoretical and contextual grounds for doing so. Third, the paradoxical low-participation pattern among highly educated urban consumers, the group with the greatest environmental knowledge and the most vocal concerns about operator legitimacy, has not been systematically explained in the DRS literature. The EBCE model addresses all three gaps through a validated structural model and a complementary qualitative investigation.

## Theoretical framework and research hypotheses

### The Extended behavioural circular economy (EBCE) model

The EBCE model is theoretically grounded in three complementary frameworks: Breckler’s24 three-component attitude model (3CAM) supplies the affective-cognitive-conative architecture; Poortinga and Pidgeon’s15 institutional trust model introduces the trust-moderated compliance mechanism; and TAM derivatives28 motivate the inclusion of perceived convenience as an independent system-accessibility predictor. This combination is appropriate for an early-stage commercially operated DRS where attitude alone is insufficient without accounting for operator trust and physical accessibility. The EBCE model integrates five latent constructs, Affective Attitudes (AA), Cognitive Attitudes (CA), Institutional Trust (IT), Perceived Convenience (PC), and Conative Attitudes and Behavioural Intention (CABI), as antecedents of Actual System Use (USE). The model is anchored in Breckler’s24 3CAM, extends it with institutional trust following Poortinga and Pidgeon^15^, and incorporates perceived convenience as an independent predictor of system use consistent with Technology Acceptance Model (TAM) derivatives^28^. Each construct was operationalised using adapted validated scales as described in the Methods section.

Affective Attitudes capture the emotional evaluative response to the REpont system, encompassing feelings of pride, environmental satisfaction, and enjoyment associated with container return. Cognitive Attitudes reflect knowledge-based evaluations of system benefits, understanding of the deposit mechanism, and awareness of environmental impacts. Institutional Trust operationalises consumer confidence in MOHU’s competence, fairness, and mission alignment. Perceived Convenience captures perceptions of RVM proximity, functionality, queue time, and ease of use. Conative Attitudes and Behavioural Intention represent the readiness to act, formed habit, and expressed intention to use the system. Self-Reported System Use (USE) is the endogenous outcome variable, measured as self-reported return frequency. Readers should note that this measure reflects participant recall rather than objective transaction data; the label “Actual Use” is retained in tables for consistency with the survey instrument but should be understood as self-reported use.

### Research hypotheses

#### H1

More positive Affective Attitudes towards the REpont system are associated with higher Cognitive Attitude scores, reflecting a sequential emotional-to-rational appraisal process in which positive emotional engagement with the system promotes more elaborate knowledge-seeking and belief formation.

This hypothesis follows directly from the affective primacy hypothesis in attitude research^29^, which posits that emotional evaluations precede and shape cognitive elaboration. In DRS contexts, consumers who feel positively about container return, experiencing it as environmentally meaningful or personally satisfying, are motivated to develop more detailed cognitive frameworks about system mechanics and benefits.

#### H2

Cognitive Attitudes exert a stronger direct effect on Conative Behavioural Intention than Affective Attitudes, consistent with the instrumental character of DRS engagement in which system knowledge drives planned participation more strongly than emotional appraisal alone.

The literature on planned behaviour in recycling contexts^20,21^ consistently shows that cognitive evaluations dominate intention formation in instrumental, goal-directed behaviours such as DRS use, where the consumer must actively plan the logistics of container collection, storage, and return. Affective attitudes are expected to operate primarily through their upstream influence on cognitive elaboration.

#### H3

Cognitive Attitudes have a statistically significant but attenuated direct effect on Actual Use, weakened by operational barriers experienced by cognitively aware users who recognize system shortcomings.

Prior research has documented a counterintuitive negative moderation of cognitive attitudes on use when system quality is perceived as inadequate^13,14,30^. Cognitively sophisticated consumers are better equipped to identify and evaluate operational failings, such as machine malfunctions or queue lengths, meaning their knowledge advantage can paradoxically suppress use frequency when service quality is inconsistent.

#### H4

Institutional Trust positively moderates the relationship between Cognitive Attitudes and Actual Use, such that the effect of system knowledge on use frequency is substantially stronger among consumers with high trust in MOHU than among those with low trust.

This moderation hypothesis follows from Poortinga and Pidgeon’s^15^ trust-compliance model. When institutional trust is high, cognitive awareness of system benefits translates efficiently into behaviour. When trust is low, behavioural activation is suppressed even among the most informed consumers, because distrust generates doubt about whether participating will genuinely achieve the environmental outcomes attributed to the system.

#### H5

Perceived Convenience has a significant positive direct effect on Actual Use alongside Conative Behavioral Intention, such that consumers perceiving lower physical and temporal costs of return report more frequent system use.

Infrastructure accessibility represents an important practical determinant of system use. A consumer may fully intend to return containers but fail to do so if RVMs are distant, frequently malfunctioning, or associated with long queues^3,5^. This hypothesis models infrastructure accessibility as an independent practical predictor of system use alongside behavioral intention.

#### H6

There is expected demographic heterogeneity in the attitudinal-behavioural relationship, with highly educated urban residents anticipated to exhibit a value-action gap characterised by high cognitive environmental awareness coexisting with lower-than-expected use frequency. Given the multi-mechanism nature of this prediction, H6 is treated as an exploratory directional hypothesis confirmed through the convergence of cluster analysis, ANOVA, and qualitative evidence.

The value-action gap literature^31^ consistently documents greater divergence between environmental attitudes and behaviour among higher-education groups, attributed to heightened sensitivity to system quality and stronger awareness of operators’ legitimacy deficits. The urban-educated paradox is expected to be particularly pronounced in Budapest, where RVM density is lower, operator distrust is higher, and time opportunity costs are greater.

## Methods

### Research design

This study employs a sequential explanatory mixed-methods design^32^, in which a dominant quantitative phase (large-scale survey analysed via PLS-SEM) is followed by a qualitative phase (semi-structured interviews) designed to contextualise, explain, and deepen quantitative findings. This design is particularly appropriate when quantitative results reveal unexpected or paradoxical patterns, such as the low participation of highly educated urban consumers, that require interpretive elaboration. The qualitative phase was explicitly informed by QUAN results: interview guides were developed after preliminary SEM analysis to probe constructs and demographic patterns identified as theoretically salient. The mixed-methods integration occurs at the discussion stage through a narrative joint display in which quantitative paths and qualitative themes are mapped to one another.

### Survey instrument design

A structured questionnaire was developed drawing on validated scales from the 3CAM literature^24–26^, institutional trust measures adapted from Poortinga and Pidgeon^15^, and perceived convenience items adapted from Oke et al.^5^ and Davis et al.^28^. All attitudinal and trust items were rated on a five-point Likert scale (1 = Strongly Disagree, 5 = Strongly Agree). Actual System Use was measured on a five-point ordinal frequency scale (1 = Never, 2 = Less than monthly, 3 = Monthly, 4 = Two to three times per month, 5 = Weekly or more often).

Scale development followed a three-stage process. In Stage 1, a comprehensive review of DRS and recycling behaviour instruments identified 48 candidate items across all constructs. In Stage 2, a bilingual expert review panel (two environmental psychologists, one DRS policy specialist, and one professional translator) assessed content validity, cultural appropriateness, and Hungarian translation quality. The instrument was translated into Hungarian using forward-backward translation with reconciliation. In Stage 3, exploratory factor analysis (EFA) with varimax rotation on a pilot sample of *n* = 220 was conducted to identify poorly loading items. Varimax rotation was selected at this item-reduction stage to obtain a clean, interpretable initial factor structure; as this pilot EFA was used solely for item screening rather than structural inference, the orthogonality assumption does not constrain the subsequent PLS-SEM, which estimates oblique latent constructs independently. Items with loadings below 0.55 or cross-loadings above 0.35 were excluded, yielding a final instrument of 20 attitudinal items across five latent constructs plus 17 demographic and contextual items covering settlement type, distance to the nearest RVM, household composition, educational attainment, and monthly household income. The full questionnaire, including both English and Hungarian item wordings, is available in the supplementary data file (DRS_Survey_Data.xlsx, Sheet: Survey_Questionnaire).

### Data collection and sampling procedure

Primary data were collected between 24 September and 24 December 2024 via a structured online survey distributed through 47 regional Facebook communities, neighbourhood forums, and university mailing lists across all seven Hungarian NUTS-2 regions. The 47 channels were selected through a systematic procedure: regional groups were identified via keyword searches and screened for minimum membership (≥ 1,000 members) and geographic coverage. This constituted a systematic convenience sample rather than probabilistic sampling; the resulting self-selection limitation is explicitly acknowledged. Recruitment posts were allocated proportionally by region based on HCSO 2024 benchmarks (6–8 posts per NUTS-2 region). The combined aggregate membership of selected online communities exceeded 280,000 registered users. The online platform was selected given the target population’s extensive social media engagement and the methodological precedent set by prior Hungarian DRS research^33^.

Of 3,088 total responses received, 423 were excluded based on four data quality criteria applied sequentially: (1) incomplete responses (missing more than 15% of attitudinal items, *n* = 182); (2) straight-lining, defined as responding identically to all attitudinal items within a single construct (*n* = 68); (3) logically implausible demographic entries, such as age-education contradictions (*n* = 31); and (4) completion time below a validated minimum threshold of 6 min, established through pilot testing (*n* = 142). The final analytical sample comprises *n* = 2,665 valid responses.

To address the potential for voluntary response bias, the sample was post-stratified using iterative proportional fitting (raking) against population distribution benchmarks from the Hungarian Central Statistical Office (HCSO, 2024)^34^ on four dimensions: gender, age group, educational attainment, and NUTS-2 region. Raking was implemented using the anesrake package in R^35^. The post-stratification procedure achieved near-perfect benchmark alignment across all strata, with maximum deviations of 0.4% points. Table 1 presents the weighted sample distribution alongside HCSO population benchmarks.

**Table 1:** Post-stratification-weighted sample distribution versus HCSO 2024 Hungarian population benchmarks (*n* = 2,665).

Variable	Category	Sample % (weighted)	Population %	Deviation
**Gender**	Male	47.6%	47.6%	0.0%
	Female	52.4%	52.4%	0.0%
**Age Group**	18–29	16.1%	16.0%	+ 0.1%
	30–39	15.7%	15.7%	0.0%
	40–59	36.3%	36.3%	0.0%
	60+	31.9%	32.0%	-0.1%
**Education**	Primary	17.1%	17.1%	0.0%
	Secondary	56.7%	56.7%	0.0%
	Tertiary	26.2%	26.3%	-0.1%
**Settlement**	Rural/Village	30.7%	30.9%	-0.2%
	Town/City	42.6%	42.8%	-0.2%
	Budapest	26.7%	26.3%	+ 0.4%

HCSO = Hungarian Central Statistical Office33. Raking implemented via anesrake package in R34. Maximum deviation across all strata: 0.4% points.

### Analytical strategy: PLS-SEM

The quantitative data were analysed using variance-based Partial Least Squares Structural Equation Modelling (PLS-SEM) implemented in SmartPLS 4.1^36^ with 5,000 bootstrap resamples for significance testing. PLS-SEM was selected over covariance-based SEM (CB-SEM) for three methodological reasons: (1) the EBCE model has a predictive-exploratory rather than purely confirmatory design; (2) the Likert-scale indicators are expected to violate multivariate normality assumptions, and PLS-SEM is robust under these conditions; and (3) the model contains multiple endogenous constructs with complex indirect and moderation paths, which PLS-SEM handles efficiently^37^.

All constructs were specified as reflective, consistent with the psychometric properties of attitude and trust measures36. The H4 moderation term (CA×IT interaction) was estimated using the product-indicator approach recommended by Henseler and Chin^38^. All indicators were mean-centred prior to generating the product terms to mitigate multicollinearity. The product-indicator method was preferred over the two-stage approach because it preserves measurement error structure within single-stage estimation. The statistical significance of the H5 direct effects was assessed using bias-corrected bootstrapped confidence intervals. Common method bias was evaluated using three procedures. Harman’s single-factor test yielded a one-factor solution explaining 38.7% of total variance (below the 50% threshold) and the marker-variable technique provided no strong evidence of problematic common method variance. Additionally, all inner-model Variance Inflation Factors (VIFs) ranged from 1.031 to 2.847, all below the recommended threshold of 3.3 (Hair et al.), providing further evidence against common method variance. Kaiser-Meyer-Olkin measure of sampling adequacy = 0.923 (excellent); Bartlett’s test of sphericity: χ²(190) = 24,112, *p* < 0.001.

Measurement model assessment followed the sequence recommended by Hair et al.^36^: indicator reliability (outer loadings ≥ 0.70), internal consistency (Cronbach’s α and composite reliability ≥ 0.70), convergent validity (AVE ≥ 0.50), and discriminant validity via the Fornell-Larcker criterion and Heterotrait-Monotrait (HTMT) ratio threshold of 0.85. Effect sizes were assessed using Cohen’s f² (0.02 = small, 0.15 = medium, 0.35 = large) and predictive relevance via Q² (PLSpredict).

### Cluster analysis

To examine H6, k-means cluster analysis (k = 3, determined via the elbow criterion and silhouette coefficient analysis) was performed. Settlement type and educational attainment were dummy-coded into numeric indicators and combined with standardised latent variable scores (Institutional Trust, Perceived Convenience) to form a richer input feature matrix, ensuring Euclidean distance metrics are applied appropriately. One-way ANOVAs with Bonferroni post-hoc tests were used to assess between-cluster differences. The optimal cluster solution was validated using the Calinski-Harabasz criterion.

### Qualitative phase: semi-structured interviews

Twenty-two semi-structured interviews were conducted between January and March 2025 with three purposively sampled stakeholder groups: consumers (*n* = 12, selected to ensure variation in age, educational attainment, settlement type, and REpont usage frequency), retail employees at supermarkets hosting RVMs (*n* = 6), and system operators or logistics staff (*n* = 4). Interview participants were recruited through purposive snowball sampling following preliminary SEM analysis. Quantitative data collection closed on 24 December 2024; data cleaning and cluster solutions were fully finalised before qualitative recruitment commenced in January 2025, ensuring the purposive sampling frame was informed by completed quantitative results. Participants targeted the high-awareness, low-participation Cluster 2 profile.

All interviews were conducted in Hungarian, lasted between 35 and 75 min (M = 52 min), and were audio-recorded with informed written consent. Transcripts were produced verbatim in Hungarian. Initial thematic coding was conducted on the original Hungarian transcripts to preserve linguistic nuance. Coded excerpts were subsequently translated into English by the author, with English-language refinement assisted by Dr Zsófia Hanjal and DeepL. Analysis followed a codebook thematic analysis approach^39^, which combines systematic codebook procedures with interpretive practice and is epistemologically compatible with reliability metrics, supported by ATLAS.ti 24. Codebook development proceeded iteratively: initial deductive codes were derived from the EBCE model constructs, and inductive codes emerged from close reading of the data. The author and an independent second coder, Dr Waqas Mazhar, coded all transcripts using the agreed codebook; intercoder reliability was κ = 0.81, indicating substantial agreement. Anonymised interview transcripts are available in the supplementary file DRS_Interview_Transcripts.docx.

### Ethical considerations

All participants provided informed written consent prior to participation consistent with the Declaration of Helsinki. Participants were assured of anonymity; all identifying information was removed from transcripts and survey data. Survey participation was voluntary with no compensation. Interview participants were informed of the study’s purpose, the use of audio recording, and their right to withdraw at any point without consequence.

## Results

### Measurement model validation

Table 2 presents the reliability and convergent validity indicators for all five latent constructs. Cronbach’s α ranged from 0.790 (CABI) to 0.919 (CA), and composite reliability (ρC) ranged from 0.823 (CABI) to 0.935 (CA), both exceeding the 0.70 threshold for all constructs. Dijkstra-Henseler’s rho_A values ranged from 0.826 to 0.921, providing additional robustness confirmation. AVE values exceeded 0.50 for all five constructs, with CABI showing the lowest AVE (0.538). Outer loadings ranged from 0.60 to 0.97, marginally above the threshold, with full discriminant validity support confirming construct distinctiveness. All outer loadings exceeded 0.60, with a range of 0.60–0.97 across indicators.

**Table 2:** Measurement model: internal consistency, reliability, and convergent validity indicators (*n* = 2,665).

Construct	Items	Cronbach α	ρC	ρA	AVE	Min λ	Max λ
Affective Attitudes (AA)	4	0.892	0.916	0.905	0.732	0.78	0.97
Cognitive Attitudes (CA)	5	0.919	0.935	0.921	0.707	0.81	0.89
Institutional Trust (IT)	4	0.874	0.902	0.884	0.699	0.77	0.88
Perceived Convenience (PC)	4	0.861	0.882	0.869	0.651	0.73	0.85
Conative Att./BI (CABI)	4	0.790	0.823	0.826	0.538	0.60	0.84

AVE = Average Variance Extracted; ρC = composite reliability (Dillon-Goldstein rho); ρA = Dijkstra-Henseler rho_A; λ = outer loading range. Thresholds per Hair et al.^36^. Most indicator loadings met the 0.70 guideline; the lowest loading was 0.60 for CABI. The retained lower-loading indicator(s) were evaluated in conjunction with acceptable construct-level reliability and AVE.

Discriminant validity was assessed using both the Fornell-Larcker criterion and the HTMT ratio. Table 3 confirms that the square root of AVE exceeded inter-construct correlations for all construct pairs (Fornell-Larcker), and HTMT ratios were below 0.85 for all pairs, with bootstrapped 95% confidence intervals not including 1.0. These results provide strong evidence of discriminant validity across all construct pairs.

**Table 3:** Discriminant validity: Fornell-Larcker criterion and HTMT ratios (5,000 bootstrap resamples).

Construct Pair	Fornell-Larcker (√AVE > *r*)	HTMT Ratio	HTMT 95% CI Upper	Assessment
AA ↔ CA	0.856 > 0.633	0.631	0.640	Valid
AA ↔ IT	0.856 > 0.521	0.587	0.596	Valid
CA ↔ CABI	0.841 > 0.717	0.799	0.807	Valid
CA ↔ IT	0.841 > 0.584	0.643	0.651	Valid
IT ↔ PC	0.836 > 0.498	0.561	0.573	Valid
PC ↔ CABI	0.807 > 0.462	0.523	0.534	Valid
CABI ↔ USE	0.734 > 0.448	0.479	0.489	Valid

HTMT = Heterotrait-Monotrait Ratio. Strict criterion < 0.85; all CI upper bounds < 1.00. Full discriminant validity established across all seven construct pairs.

### Structural model and hypothesis testing

Table 4 presents the full structural model results. The model explained R² = 0.400 of variance in Cognitive Attitudes, R² = 0.638 in Conative Attitudes/BI, and R² = 0.341 in Actual Use. Model fit was assessed using the Standardised Root Mean Square Residual (SRMR = 0.063, below the 0.08 threshold). Although SRMR was originally developed for CB-SEM, Henseler et al. (2016) demonstrated its applicability to PLS-SEM under the composite model, and the value obtained confirms acceptable global fit. Predictive relevance Q² was positive for all endogenous constructs (Q²CA = 0.273; Q²CABI = 0.331; Q²USE = 0.091), indicating that the model has predictive power beyond the simple mean baseline. H1–H5 were supported or partially supported in the structural model; H6 was evaluated separately through the demographic, cluster, and qualitative analyses.

**Table 4:** PLS-SEM structural model results: standardised path coefficients, bootstrap statistics, and hypothesis outcomes (*n* = 2,665; 5,000 resamples).

Path	H	β	SE	t	*p*	95% CI	f²	Result
AA → CA	H1	0.633	0.011	57.52	< 0.001	[0.612, 0.654]	0.665	Supported
CA → CABI	H2	0.799	0.008	99.88	< 0.001	[0.783, 0.815]	1.757	Supported
CA → USE (direct)	H3	0.279	0.017	16.41	< 0.001	[0.246, 0.312]	0.089	Partial
IT → CA	H4a	0.241	0.019	12.68	< 0.001	[0.204, 0.278]	0.068	Supported
CA×IT → USE	H4b	0.183	0.022	8.32	< 0.001	[0.140, 0.226]	0.041	Supported
CABI → USE	H5a	0.268	0.018	14.89	< 0.001	[0.233, 0.303]	0.082	Supported
PC → USE	H5b	0.157	0.021	7.48	< 0.001	[0.116, 0.198]	0.029	Supported
AA → CABI (ind.)	—	0.506	0.009	56.22	< 0.001	[0.488, 0.524]	med.	Supports H1–H2 chain

β = standardised path coefficient; SE = bootstrap standard error; f² = Cohen effect size (0.02 small, 0.15 medium, 0.35 large); H3 is classified as partially supported because the CA→USE direct effect was statistically significant but modest in magnitude (β = 0.279, f² = 0.089), while the dominant structural relationship was CA→CABI. The AA→CABI indirect effect (via CA) is reported here as a robustness check on the H1–H2 chain and is not a test of H6; H6 is evaluated separately in Sect.  5.5 through demographic, cluster, and qualitative evidence. SRMR = 0.063 (acceptable fit). All estimates based on 5,000 bootstrap resamples in SmartPLS 4.1.

The dominant structural path is CA→CABI (β = 0.799, f² = 1.757), indicating a large effect and confirming H2. Affective Attitudes predict Cognitive Attitudes with a large effect (β = 0.633, f² = 0.665), supporting H1. Institutional Trust exerts a significant direct effect on Cognitive Attitudes (β = 0.241, H4a) and significantly moderates the CA→USE relationship (βinteraction = 0.183, H4b), confirming H4. Table 5 reports significant indirect effects, including the full AA→CA→CABI mediation chain (β = 0.506, CI: [0.488, 0.524]) (Fig. 1).

**Table 5:** Significant indirect and total effects (bias-corrected bootstrapping, 5,000 resamples).

Indirect Path	β	95% CI	Interpretation
AA → CABI (via CA)	0.506	[0.488, 0.524]	Affect shapes cognition, then drives intention
AA → USE (via CA)	0.177	[0.158, 0.196]	Emotional engagement drives use through understanding
AA → USE (full mediation)	0.135	[0.121, 0.149]	Sequential affective-cognitive-conative-use chain
AA → USE (total indirect)	0.224	[0.205, 0.243]	Combined emotional influence on behaviour
CA → USE (via CABI)	0.214	[0.196, 0.232]	Knowledge activates habit, which activates use
IT → USE (via CA→CABI)	0.087	[0.068, 0.106]	Trust scaffolds the cognition-to-behaviour chain

All indirect effects significant at *p* < 0.001.

### EBCE Model path diagram

**Fig. 1:**
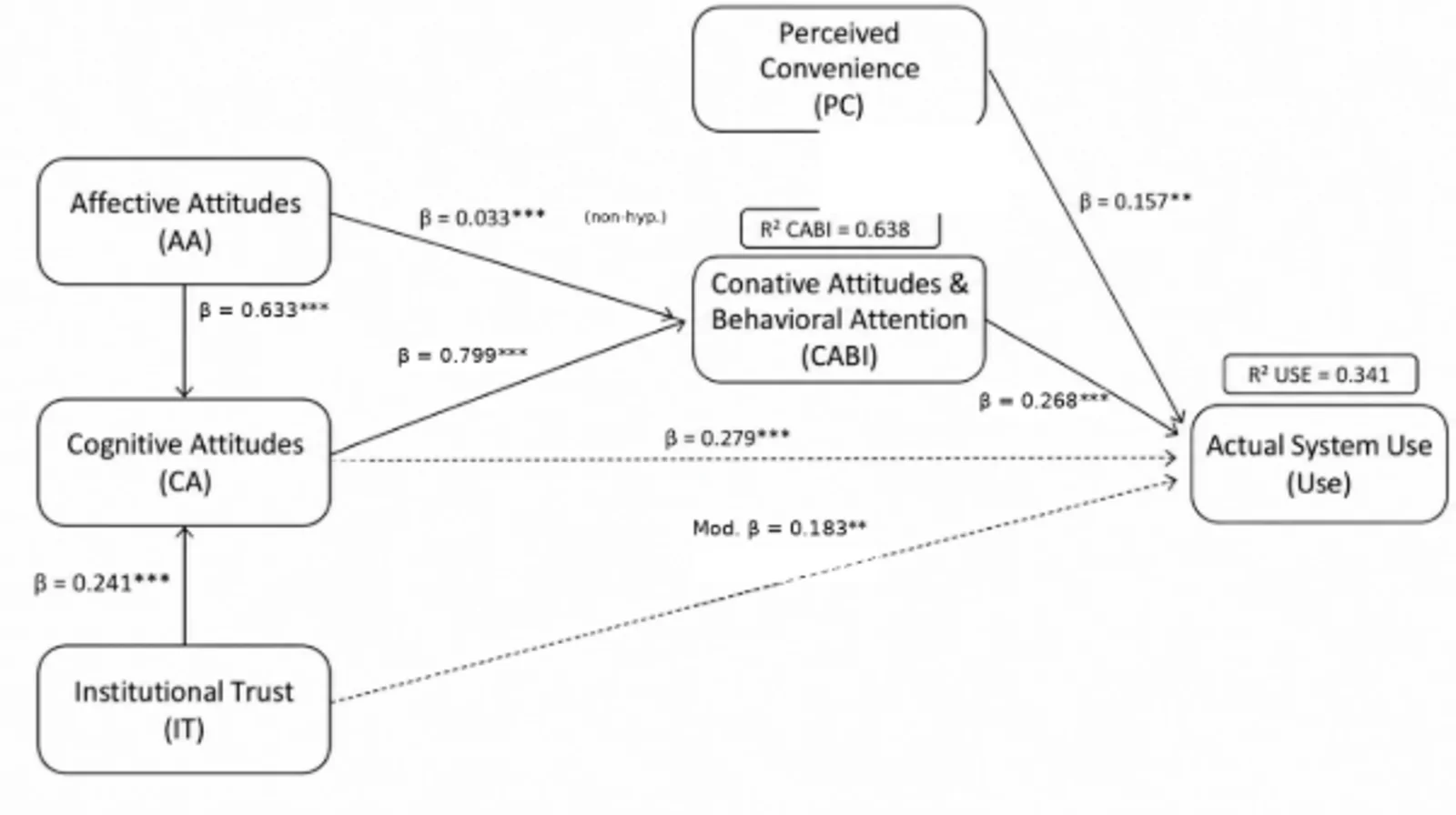
Extended Behavioural Circular Economy (EBCE) model with standardised path coefficients (β). Solid arrows indicate direct effects; dashed arrows indicate indirect or moderation paths. R² values shown for endogenous constructs. AA = Affective Attitudes; CA = Cognitive Attitudes; IT = Institutional Trust; PC = Perceived Convenience; CABI = Conative Attitudes and Behavioural Intention; USE = Actual System Use. All paths *p* < 0.01 or better.

### Moderation analysis: institutional trust

Simple slope analysis reveals a pronounced moderation of the CA→USE relationship by Institutional Trust. Among high-trust respondents (+ 1 SD above mean IT), the cognitive-to-use slope is βconditional = 0.421 (95% CI: [0.393, 0.449]). Among low-trust respondents (-1 SD), the slope falls to βconditional = 0.137 (95% CI: [0.114, 0.160]). The moderation effect size (f² = 0.041) is classified as small-to-medium in structural models. This pattern confirms H4b: cognitive awareness of REpont’s environmental benefits translates into actual use primarily when consumers trust the MOHU operator to deliver on the system’s stated purpose. Among low-trust consumers, the same awareness produces little behavioural activation, indicating that knowledge is necessary but institutionally insufficient for driving use.

### Demographic and cluster analysis

One-way ANOVA revealed significant settlement-type effects on REpont use frequency (F(2, 2662) = 7.43, *p* < 0.001, η² = 0.006). Post-hoc Bonferroni tests confirmed that Budapest residents reported significantly lower usage (M = 3.07, SD = 1.21) than both urban non-capital (M = 3.27, SD = 1.15, *p* < 0.01) and rural residents (M = 3.23, SD = 1.18, *p* < 0.05). Education effects were also significant (F(2, 2662) = 9.17, *p* < 0.001, η² = 0.007), with university graduates reporting lower usage (M = 3.15) than secondary-school respondents (M = 3.30, *p* < 0.01). The settlement×education interaction was also significant (F(4, 2660) = 4.88, *p* < 0.01, η² = 0.007), indicating that the negative effect of tertiary education on use is most pronounced in Budapest.

K-means cluster analysis (k = 3) identified three consumer profiles with meaningfully distinct attitudinal and behavioural characteristics (Table 6). Cluster 1 (Rural/Secondary education, *n* = 794, 29.8%) represents conventional participants with moderate cognitive and institutional trust scores and average use frequency. Cluster 3 (Urban/Secondary education, *n* = 1,099, 41.2%) represents the mainstream participant group with the highest use frequency and balanced attitudinal profile. Cluster 2 (Urban-Capital/University, *n* = 772, 29.0%) constitutes the most theoretically significant finding: despite exhibiting the highest mean Cognitive Attitude scores (M = 4.02) and similar Affective Attitude scores to other clusters (M = 4.08), this group reports the lowest Actual Use (M = 3.10), the lowest Institutional Trust (M = 2.71), and the lowest Perceived Convenience (M = 2.88). This pattern constitutes a robust empirical manifestation of the value-action gap predicted by H6.

**Table 6:** Consumer cluster profiles: mean attitudinal and behavioural variables with between-cluster significance tests.

Cluster	Profile	*N*	M USE	M CA	M AA	M IT	M PC
C1	Rural / Secondary	794	3.23	3.74	4.12	3.18	3.42
C2	Urban-Capital / Univ.	772	3.10*	4.02	4.08	2.71*	2.88*
C3	Urban / Secondary	1,099	3.28	3.81	4.18	3.22	3.38

* Significantly different from the other two clusters (Bonferroni post-hoc, *p* < 0.05). M = mean; USE: 1 = Never, 5 = Always/Weekly. CA = Cognitive Attitudes; AA = Affective Attitudes; IT = Institutional Trust; PC = Perceived Convenience. The Cluster 2 paradox: highest CA coexists with lowest IT, PC, and USE.

### Qualitative findings: thematic analysis

Codebook thematic analysis of 22 interviews (κ = 0.81) yielded four major themes, each comprising two to four sub-themes. The themes contextualise and extend the quantitative findings by revealing social, cultural, and institutional mechanisms that survey instruments cannot capture.

***Theme 1: Infrastructure Friction and RVM Availability***

Coded in 19 of 22 interviews, this theme captures systematic consumer frustration with RVM availability, malfunction rates, and queuing dynamics. Three sub-themes emerged: machine unavailability as deterrent, queue time as opportunity cost, and spatial distribution inequity. The machine unavailability sub-theme was most prevalent in central Budapest, where population density and limited retail floor space restrict RVM installation. Retail employee respondents independently confirmed that single-machine stores generate regular queue overflow that deters returning shoppers.

*“I’ve gone to the Lidl three times this week and the machine was broken every single time. After the third time I just threw the bottles in the regular recycling. I know it’s not the same*,* but what am I supposed to do?”*

Consumer, female, 34, Budapest, Graduate degree (Cluster 2).

*“We have one machine for the whole store. On weekends the queue is 15 to 20 people. People who came to shop in five minutes are not going to stand in line for bottles. They just give up.”*

Retail employee, Tesco, District VIII Budapest.

Operator respondents acknowledged infrastructure density as the primary constraint on participation growth in the first year of operation, with a planned RVM expansion program expected to significantly increase urban coverage in 2025–2026. This theme provides the primary qualitative grounding for the Perceived Convenience direct effect (H5) and suggests that infrastructure expansion is a high-priority policy intervention, consistent with the quantitative modelling results.

***Theme 2: Social Stigma and the Marginalisation Effect***

Eleven of 12 consumer interviewees spontaneously raised the social dynamics at return points as a factor influencing their participation. A consistent pattern emerged: the regular presence of unhoused individuals and informal bottle collectors at or near RVM stations creates a social environment that educated urban consumers perceive as uncomfortable or socially inappropriate, deterring use even when the consumer is cognitively aware that their reaction is not rationally justified. This pattern resonates with the stigmatised redemption space literature^40^ and provides a qualitative mechanism for the Cluster 2 institutional trust deficit: the return space is experienced as institutionally exclusionary, reducing perceived system legitimacy.

*“I feel bad saying this*,* but there’s always a group of people collecting bottles around the machine and I feel. strange standing there. Like it’s not for me*,* even though I know intellectually that’s not right. It feels marginalised.”*

Consumer, male, 41, Budapest, University-educated (Cluster 2).

*“We see this every week. Some regular customers*,* professional people*,* they go to the machine*,* they see the queue and the situation*,* and they walk away. They come back another time*,* or they don’t.”*

Retail employee, Aldi, District XIV Budapest.

***Theme 3: Cultural Bottle Accumulation Behaviour***

All 12 consumer interviewees described a culturally embedded practice of accumulating containers at home, typically in a dedicated bag, box, or pantry space, for eventual bulk return. This “bottle-hoarding” behaviour represents a temporal decoupling of the financial incentive from the return behaviour. Unlike Nordic contexts where container return is seamlessly integrated into the weekly shopping trip^3,4^, Hungarian consumers tend to accumulate until storage pressure prompts action, sometimes over periods of weeks or months. This pattern reduces return frequency, explains why the 50 HUF deposit does not reliably activate weekly behaviour, and constitutes a cultural behavioural mediator not captured by the quantitative instrument.

*“We have a big bag in the pantry. When it’s full we go. But sometimes the bag is full for two months and then we just forget. The 50 forint per bottle is not enough to make us go out of the way*,* it has to be already on our route*,* already in the shopping trip.”*

Consumer, female, 52, Town, Secondary education (Cluster 1).

*“Even people who are enthusiastic about the environment*,* they accumulate bottles at home. I do it too. You need a system*,* like*,* the bag is always in the car*,* not in the kitchen.”*

Consumer, male, 38, Budapest, Postgraduate (Cluster 2).

***Theme 4: Operator Legitimacy and Concession Model Distrust***

Distrust of the MOHU concession operator was a recurrent theme, particularly among urban and tertiary-educated respondents. Multiple interviewees articulated systemic scepticism about MOHU’s environmental mission, grounded in three distinct concerns: MOHU’s ownership by MOL Group, Hungary’s dominant oil and gas conglomerate; uncertainty about whether returned materials genuinely reach recycling facilities or are diverted to landfill or incineration; and perceived unfairness of the concession arrangement, whereby a commercial entity captures the financial value of unclaimed deposits. This theme provides the strongest qualitative explanation for the institutional trust deficit in Cluster 2 and the moderation of the CA→USE path observed in the structural model.

*“MOHU is MOL*,* a petrochemical company. I return my plastic bottle to a company that produces plastic. It feels circular in a way that doesn’t benefit the environment. I wonder where the bottles really go.”*

Consumer, male, 29, Budapest, Postgraduate degree (Cluster 2).

*“I tell customers the bottles go to recycling. But honestly I don’t know where they end up. We put them in containers and MOHU collects them. After that it’s a black box.”*

Retail employee, Lidl, District VII Budapest.

## Discussion

### The sequential attitude-behaviour architecture

The EBCE model’s dominant structural finding, the large-effect CA→CABI path (β = 0.799, f² = 1.757), establishes that cognitive system evaluations primarily operate through behavioural intention formation and habitual commitment rather than direct behavioural activation. This finding has important practical implications: interventions that successfully shift cognitive attitudes, through information provision or system transparency, should be expected to produce stronger effects on intention formation and sustained habit development than on immediate use frequency. The AA→CA path (β = 0.633) confirms that emotional engagement is an upstream driver of cognitive elaboration, consistent with the affective primacy hypothesis^29^. This means that campaigns leading with emotional resonance, pride in local environmental action, visible community impact, and positive social identity, can effectively prime cognitive processing, whereas campaigns leading with technical system information will have limited effect in the absence of prior affective engagement.

### Institutional trust as the critical behavioural gateway

The institutional trust moderation represents the study’s most theoretically novel and practically consequential finding. The conditional effect analysis reveals a threefold difference in the CA→USE slope between high-trust (β = 0.421) and low-trust (β = 0.137) consumers. This magnitude of moderation implies that operator legitimacy is not a peripheral concern but a structural gateway determining whether the entire attitude-to-behaviour conversion mechanism functions. The qualitative data explain the mechanism with unusual clarity: MOHU’s association with a petrochemical parent company creates a mission-alignment deficit that suppresses behavioural activation even among consumers whose cognitive attitudes and environmental values are strongly positive.

This finding extends Poortinga and Pidgeon’s15 trust-compliance model to circular economy infrastructure. It is important to note that the CA→USE direct path remains statistically significant (β = 0.279, *p* < 0.001), confirming that cognitive attitudes predict use independently; the institutional trust moderation amplifies this effect substantially among high-trust consumers. In environmental risk regulation, low institutional trust reduces policy compliance; in DRS contexts, the same mechanism reduces behavioural uptake of a voluntary incentive system. The implication is that DRS performance is not solely a function of deposit level and infrastructure density, as the operational literature suggests1,3, but depends critically on consumers’ beliefs about the operator’s integrity and mission alignment. This is a dimension that system design and communication strategy must explicitly address.

### The urban-educated paradox: a structural explanation

The Cluster 2 paradox, the highest cognitive awareness combined with the lowest institutional trust and lowest actual use, provides empirically robust support for the value-action gap hypothesis (H6). The qualitative investigation reveals that this pattern is not simply a gap between knowledge and behaviour, but reflects a specific configuration of three reinforcing barriers: social stigma at return points that reduces perceived system inclusivity, operator legitimacy concerns grounded in political economy awareness, and high opportunity cost of time in dense urban environments where RVM queues impose disproportionate temporal burdens on professional workers. These three barriers jointly suppress behavioural activation in the group most predisposed to environmental action on cognitive grounds, consistent with what may be termed an “informed abstention” pattern, though longitudinal data would be needed to confirm deliberate non-participation as the operative mechanism.

This explanation has important implications for DRS policy. It suggests that the value-action gap in this context is not a psychological deficit requiring attitude change, but a rational response to institutional conditions. Interventions targeting this group cannot operate through the cognitive channel, which is saturated, but must address the social environment of return (Theme 2), operator transparency (Theme 4), and infrastructure convenience (Theme 1). Critically, the moderate R² for USE (0.341) indicates that unmeasured variables, including social norms, household routines, and spatial factors not captured in the survey, explain substantial additional variance in actual use, and these factors are likely disproportionately operative in the Cluster 2 context.

### Cultural bottle accumulation as a behavioural mediator

The culturally embedded bottle-accumulation practice identified in Theme 3 represents a previously undocumented mediation of the deposit incentive in Central European DRS contexts. This behaviour is structurally distinct from the return-on-shopping-trip integration observed in mature Nordic systems. It implies that the deposit’s behavioural effect is not continuous but batch-activated, triggered by storage pressure rather than routine integration into the weekly shopping schedule. This pattern has two policy implications: first, interventions that facilitate storage and transport, such as compact collection bags or in-car container holders distributed at point of sale, may increase return frequency by reducing friction at the storage-to-return transition; second, digital tools such as deposit balance tracking in the MOHU mobile application may increase the salience of accumulated deposits and nudge more frequent returns.

## Policy implications

The EBCE model findings generate actionable recommendations across four policy domains.

Infrastructure investment and RVM density. The Perceived Convenience direct effect (H5) and Theme 1 collectively identify RVM expansion in inner-city Budapest as a high-priority infrastructure investment, with converging quantitative and qualitative evidence suggesting substantial potential to increase participation rates. The significant negative effect of Budapest residence on use frequency, combined with qualitative evidence of machine unavailability and queue deterrence, indicates that infrastructure constraints are suppressing behavioural intentions that would otherwise translate into use. The REpont operator’s planned 2025–2026 expansion programme should prioritise neighbourhood-level, non-retail RVM deployment, such as in libraries, transport hubs, and post offices, particularly in districts with high tertiary-educated populations and low current RVM density.

Deposit level and incentive design. The 50 HUF deposit appears sufficient to initiate participation but insufficient to sustain weekly return frequency without accompanying convenience improvements. International evidence^3^ consistently shows a positive correlation between deposit level and return rate, with higher deposits particularly effective in stimulating participation among lower-income groups. A graduated deposit increase to 100 HUF, or gamified donation options allowing consumers to redirect deposit value to environmental charities, could strengthen behavioural anchoring and increase return frequency among the accumulated-bottles cluster.

**Operator communication and legitimacy.** The institutional trust moderation (H4b) and Theme 4 together constitute the strongest evidence that MOHU’s operator communication strategy requires fundamental revision. Trust-building measures should include: independent third-party auditing of material recycling pathways with publicly reported results; transparent disclosure of where recycled materials are sold and what environmental value is generated; and communication campaigns that explicitly address the mission-alignment concerns identified in the interviews. The language of these campaigns should be specific and verifiable rather than aspirational, as the Cluster 2 group is demonstrably sceptical of generic environmental claims.

**Social inclusion and return environment design.** The social stigma documented in Theme 2 is both an equity issue and a participation barrier. Dedicated non-commercial RVM locations in public institutions would remove the social context that deters educated urban users. Inclusive RVM design, such as accessible interfaces, multilingual instructions, and clean, well-lit return environments, can simultaneously reduce barriers for elderly and disabled users and normalise the return experience for higher-income groups. Public communication emphasising the mainstream and cross-demographic nature of REpont participation, by displaying aggregate community return statistics, can counteract the stigmatisation dynamic.

## Limitations and future research

Several limitations of this study warrant acknowledgment. First, the online recruitment strategy introduces self-selection bias (attitudinally engaged respondents) and digital exclusion bias (non-internet users, disproportionately elderly and low-income groups). Although post-stratification weighting reduces observable demographic imbalance, residual attitudinal selection bias cannot be fully corrected by weighting. Results should therefore be interpreted as representative of the digitally connected Hungarian adult population rather than the full population. Replication studies should consider CAPI methods to reach lower-education and older groups more representatively.

Second, the cross-sectional survey design precludes temporal causal inference. The sequential attitude-behaviour architecture confirmed by PLS-SEM is theoretically motivated and statistically consistent, but establishing temporal precedence requires longitudinal data. Future research should deploy panel designs tracking the same respondents across multiple REpont participation seasons, ideally with access to actual transaction data from REpont accounts to provide objective behavioural measures beyond self-report.

Third, the qualitative sample (*n* = 22), while purposively designed to cover variation across stakeholder groups, settlement types, educational levels, and DRS usage patterns, cannot claim statistical representativeness. Qualitative findings should be interpreted as theoretical elaboration and mechanistic explanation of quantitative patterns, not as prevalence estimates. Larger qualitative studies with maximum variation sampling could strengthen the generalisability of the thematic findings.

Fourth, the product-indicator moderation approach for H4 provides a reliable estimate of the linear moderation effect but may not fully capture non-linear trust dynamics. Future research should explore quadratic and threshold effects of trust on the attitude-behaviour relationship using response surface analysis.

Fifth, the EBCE model does not capture habit strength as a distinct construct, relying instead on the conative component of the 3CAM as a proxy for habitual readiness. Future model extensions should incorporate an explicit habit measure, such as Verplanken and Orbell’s^41^ Self-Report Habit Index, to distinguish between intentional and automatised participation. Finally, comparative studies across new EU DRS adopters, including Romania, Ireland, Poland, and Croatia, will be essential to distinguish context-specific from cross-nationally generalizable behavioural mechanisms in early-stage systems.

## Conclusion

This study has proposed and empirically tested the Extended Behavioural Circular Economy (EBCE) model, a five-construct PLS-SEM framework that integrates the three-component attitude model with institutional trust and perceived convenience, applied to consumer participation in Hungary’s REpont DRS. Validated on a nationally post-stratification-weighted sample of *n* = 2,665 and enriched by 22 semi-structured stakeholder interviews, the model indicates a sequential affective-to-cognitive-to-conative-to-use predictive architecture and identifies institutional trust as a critical moderating gateway: the cognitive-to-use conversion is threefold stronger among high-trust consumers than among low-trust consumers, regardless of attitude levels.

The study makes three principal theoretical contributions. First, to the author’s knowledge this represents a rare empirical test of institutional trust as a formally specified moderating construct in DRS research, with results indicating that operator legitimacy functions as an important condition for efficient attitude-to-behaviour translation. Second, it identifies perceived convenience as an independent direct predictor of system use alongside conative behavioral intention, demonstrating the importance of infrastructure accessibility in explaining participation. Third, the value-action gap documented in Cluster 2 is consistent not with a psychological deficit but with responses to specific institutional and infrastructural conditions, including operator mission-alignment concerns, social environment quality at return points, and urban infrastructure constraints, a structural explanation with direct policy relevance.

As the PPWR mandates DRS adoption across most EU member states by 2029, the Hungarian experience offers potentially informative lessons that warrant cross-national validation for policymakers and system designers in transitioning economies. Behavioural adoption lags infrastructure deployment in early-stage systems. The most environmentally aware citizens may be paradoxically the hardest to retain as regular users when system quality falls short of their expectations. And institutional trust, grounded in perceived operator integrity and mission alignment, is not a soft reputational concern but a significant moderating condition in the present model, shaping whether financial incentives and environmental attitudes translate into the sustained participation that makes DRS systems environmentally effective. Addressing this trust dimension should be as central to DRS design as deposit levels and machine deployment.

## Data Availability

Survey raw data, descriptive statistics, PLS-SEM results, measurement model indicators, cluster profiles, and the full survey questionnaire (English and Hungarian) are provided in the supplementary file DRS_Survey_Data.xlsx. Anonymised qualitative interview transcripts are available in DRS_Interview_Transcripts.docx. Both files are available upon publication or on reasonable request to the corresponding author.

## Code availability

No custom software, bespoke computational tool, new algorithm, or original computational pipeline was developed for this study. The analyses were conducted using established software and packages described in the Methods.
